# Calcium-Binding Protein S100P Promotes Tumor Progression but Enhances Chemosensitivity in Breast Cancer

**DOI:** 10.3389/fonc.2020.566302

**Published:** 2020-09-15

**Authors:** Yizi Cong, Yuxin Cui, Suxia Wang, Lei Jiang, Jianqiao Cao, Shiguang Zhu, Emily Birkin, Jane Lane, Fiona Ruge, Wen G. Jiang, Guangdong Qiao

**Affiliations:** ^1^Department of Breast Surgery, The Affiliated Yantai Yuhuangding Hospital of Qingdao University, Yantai, China; ^2^Cardiff China Medical Research Collaborative, School of Medicine, Cardiff University, Cardiff, United Kingdom; ^3^Department of Pathology, The Affiliated Yantai Yuhuangding Hospital of Qingdao University, Yantai, China

**Keywords:** breast neoplasms, S100P, tumor progression, chemosensitivity, HER2

## Abstract

**Background:**

Chemoresistance remains one of the obstacles to overcome in the treatment of breast cancer. S100 calcium-binding protein P (S100P) has been observed to be overexpressed in several cancers and has been associated with drug resistance, metastasis, and prognosis. However, the role of S100P in chemoresistance in breast cancer has not been thoroughly determined.

**Methods:**

Immunohistochemistry was used to evaluate the expression level of S100P protein in 22 pairs (pre-chemo and post-chemo) of breast cancer tissue from patients who underwent neoadjuvant chemotherapy. The influence of S100P on the biological behavior and chemosensitivity of breast cancer cells was then investigated.

**Results:**

The protein level of S100P in breast cancer tissue was significantly higher than in benign fibroadenoma (*p* < 0.001). The S100P expression level was shown to be decreased by 46.55% after neoadjuvant chemotherapy (*p* = 0.015). Subgroup analysis revealed that S100P reduction (57.58%) was mainly observed in the HER2+ tumors (*p* = 0.027). Our *in vitro* experiments showed that the knockdown of S100P suppressed the proliferation, adhesion, migrative and invasive abilities of T47D and SK-BR-3 breast cancer cells. We further demonstrated that this knockdown increased the chemoresistance to paclitaxel and cisplatin in SK-BR-3 cells. We found S100P exerted its function by upregulating NF-κB, CCND1 and Vimentin, but downregulating E-cadherin.

**Conclusion:**

S100P promotes the aggressive properties of breast cancer cells and may be considered as a promising therapeutic target. Moreover, S100P can be used to predict the therapeutic effect of chemotherapy in HER2+ breast cancer patients.

## Introduction

Breast cancer is the most common malignancy and the second leading cause of cancer death in women (1). Chemotherapy is widely applied to improve the survival of patients with breast cancer. However, some patients inevitably manifest chemoresistance in either an intrinsic or an acquired manner. It has been a great challenge to tackle this problem for the better treatments of those breast cancer patients. This is particularly the case for patients with triple-negative breast cancer (TNBC) type or metastasis because the response to chemotherapeutic drugs may be vital for them to survive (2). Therefore, it is a prerequisite to unveil the complicated mechanisms underlying the chemoresistance before better treatment could be developed to fulfill the need clinically.

The S100 family consists of more than 20 small dimeric Ca^2+^-binding proteins, and is the largest group of the helix-loop-helix (EF-hand) superfamily (3). S100 proteins are found to be involved in the regulation of calcium homeostasis, cell proliferation and apoptosis, cell invasion and motility, cancer metastasis, angiogenesis, cytoskeleton interactions, protein phosphorylation, regulation of transcriptional factors, autoimmunity, chemotaxis, and inflammation (4). S100P was originally identified in the human placenta (5) and is also expressed in other organs such as the stomach, urinary bladder, and bone marrow (6). It contains a characteristic structural domain known as the EF-hand motif, which exists as intracellular or secreted homo- or hetero-dimers with composition depending on the cellular context (7). The functions of S100P are mainly attributed to its interaction with or regulation of several molecules that regulate actin cytoskeleton dynamics and extracellular matrix remodeling, including Ezrin, IQGAP1, myosin IIA, cathepsin D, and phosphorylated cofilin (8–11). S100P acts as a signaling molecule in intracellular components and the extracellular matrix (12, 13). Although the mechanisms that regulate S100P have not been fully documented, DNA microarray indicates that S100P is upregulated by estradiol (14), progesterone (15), and HER2 overexpression (16), which is in line with the clinical findings that high S100P levels are related to ER/PR and HER2 overexpressing tumors. The significant association between S100P and ER expression implies that S100P is involved in the early stages of breast carcinogenesis (17). S100P is also linked to the immortalization of breast epithelial cells *in vitro*, tumor progression and early relapse in patients (17–19).

S100P has recently attracted great attention due to its implication in malignant transformation and tumor progression, and in predicting prognosis and metastasis in several cancer types (20). The implication of S100P in the carcinogenesis and progression of breast cancer has also been reported (17, 19). S100P expression is elevated in TNBC tissues (21) and associated with poor survival of the TNBC patients (22). TNBC patients with the low cytoplasmic levels of both S100P and Ezrin have been shown to confer a better disease-free survival (DFS) compared to other TNBC patients (23). S100P is thought to exerts its oncogenic activities via the activation of receptor for advanced glycation end products (RAGE) (24). The extracellular ligand-binding domain of RAGE can bind to several ligands, including S100P, to initiate downstream signaling pathways that promote cell proliferation, viability and motility. Blocking S100P interaction with RAGE is sufficient to inhibit the growth of tumors (25).

Additionally, S100P dimers, formed in response to the increase in cellular calcium concentrations, can bind and activate the cytoplasmic protein Ezrin (7). This interaction promotes trans-endothelial migration (TEM) in patients with lung cancer, pancreatic cancer, and TNBC (23). Moreover, S100P enhances cell proliferation by upregulating cyclin D1 and CDK2 in human hepatocellular carcinoma (26). NORAD overexpression in the TNBC cell line MDA-MB-231 can block the interaction of S100P with IQGAP1 and p53, leading to the downregulation of cathepsin D and a reduction in cofilin phosphorylation (27). The protein accumulation of S100P at tumor sites also stimulates tumor invasion by inducing angiogenesis (28). Although S100P is mainly located in the cell nucleus and cytoplasm, it can also be secreted into the extracellular matrix in an autocrine or paracrine manner (29). High plasma levels of S100P correlate robustly with poor prognosis of metastatic breast cancer (MBC) patients. In addition, the plasma level of S100P reduced significantly in response to the radiographic treatment of these patients (30).

S100P is also involved in drug response in different cancer types, either by increasing chemoresistance (9, 31) or enhancing chemosensitivity (32, 33). S100P overexpression is related to the resistance to 5-fluorouracil in pancreatic cancer (9) and irinotecan in prostate cancer (34). There is a correlation between overexpression of S100P and resistance to cyclophosphamide, etoposide, methotrexate, and mitoxantrone in different cancer cell lines (31). S100P also binds p53, together with its negative regulator HDM2, and perturbs the p53-HDM2 complex binding and increases the p53 level. However, the S100P-induced p53 is not able to activate its transcriptional targets (e.g., hdm2, p21WAF, and bax) following the DNA damage and enhances chemoresistance by binding and inactivating p53 (35). In contrast to the above findings, studies on ovarian cancer cells show a chemo-sensitization effect of S100P in response to drugs including carboplatin and paclitaxel, 5-fluorouracil, etoposide, and doxorubicin (32). Similarly, studies on the downregulation of S100P in colon cancer cell line 8307 suggest that S100P is associated with oxaliplatin sensitivity in the drug-resistant cells (33). These findings suggest that either drug resistance or sensitivity may be regulated by S100P in different cancers.

Until now the role of S100P in the response of breast cancer to chemotherapeutic drugs remains unclear. In this study, we collected breast tissue samples from 22 pairs (pre-chemo and post-chemo) of breast cancer tissues from patients who underwent neoadjuvant chemotherapy. Immunohistochemistry was used to investigate expression levels of S100P protein, and changes between pre-chemotherapy and post-chemotherapy were analyzed. Using T47D and SK-BR-3 breast cancer cell lines, the influence of S100P on cell behavior, biological function, and chemosensitivity was explored *in vitro*.

## Materials and Methods

### Cell Lines and Culture

The human breast cancer cell lines including T47D and SK-BR-3 were obtained from ATCC (Middlesex, United Kingdom). The cells were subcultured at 37°C, 5% CO_2_ in Dulbecco’s Modified Eagle’s Medium (DMEM/Ham’s F-12 with L-Glutamine) (Sigma-Aldrich, Dorset, United Kingdom) supplemented with 1× antibiotics penicillin and streptomycin (Sigma-Aldrich, Dorset, United Kingdom) and 10% fetal bovine serum (FBS) (Sigma-Aldrich, Dorset, United Kingdom).

### Stable Cell Lines With S100P Knockdown

To establish the stable S100P knockdown breast cancer cell lines, lentiviral-derived vectors containing S100P shRNA[pLV(shRNA)-EGFP:T2A:Puro-U6>hS100P(shRNA#1)] or Scramble shRNA (Scr) negative control [pLV(shRNA)- EGFP:T2A:Puro-U6>Scramble_shRNA] were transfected into T47D and SK-BR-3 cells (Vector builder, United States) based on the manufacturer’s protocol. Briefly, 5×10^4^ cells were loaded into a 6-well plate, incubated overnight, and changed to medium with 10μg/ml of polybrene and lentiviral particles. After incubation for 20 h, normal medium was used for subsequent culture for 3 days. Puromycin (Sigma, St. Louis, MO, United States), at a concentration of 2 μg/ml, was applied for the specific selection of the stable cells. After selection for 1 week, the stable cells were cultured in normal medium with the addition of 0.25 μg/ml puromycin for the maintenance of the stable cell property.

### Quantitative Real-Time PCR (q-PCR)

Total RNA was extracted from cultured cells using TRIzol reagent (Sigma-Aldrich, Dorset, United Kingdom) following the manufacturer’s instructions. RNA was reverse transcribed into cDNA using the GoScript^TM^ Reverse Transcription System kit (Promega, Madison, WI, United States). The quantitative real-time PCR was performed with an iCycler iQ (Bio-Rad Laboratories, Hemel Hempstead, United Kingdom) following the cycling conditions: 94°C for 5 min, 100 cycles of 94°C for 10 s, 55°C for 35 s and 72°C for 20 s. The primer sequences of S100P were: F: ATCATAGACGTCTTTTCCCG; zR: **ACTGAACCTGACCGTACA** CACTTGAGCAATTTATCC ACGG (Z sequence is highlighted in bold font). The mRNA levels were normalized to those of glyceraldehyde-3-phosphate dehydrogenase (GAPDH) using the method of 2^–Δ^ ^*Ct*^.

### Western Blotting

Cultured cells were detached and lysed with a protein lysis buffer. Protein concentration was measured using a Bio-Rad DC protein assay kit (Hemel-Hempstead, United Kingdom). Equal amounts of protein samples were separated by sodium dodecyl sulfate-polyacrylamide gel electrophoresis (SDS-PAGE) and blotted to PVDF membrane. The membrane was then blocked with 5% skimmed milk for 2 h. Proteins were specifically probed with a primary antibody and peroxidase-conjugated secondary antibody respectively. Protein signals were visualized with a Luminata Forte Western HRP substrate (Merck Millipore, Hertfordshire, United Kingdom) and assessed by ImageJ software (National Institutes of Health, Bethesda, MD, United States) based on the intensity of the blotted bands.

The antibodies of S100P (#ab133554), NF-κB (p65) (#ab16502), and Vimentin (#ab137321) were acquired from Abcam (Cambridge, United Kingdom). GAPDH (#sc-47724), CCND1 (#sc-8396) antibodies were obtained from Santa Cruz (Insight Biotechnology Limited, Middlesex, United Kingdom). E-cadherin (#AF748) was purchased from R&D Systems (Abingdon, Oxfordshire, United Kingdom). Anti-mouse (#A5278), anti-rabbit (#A0545) secondary antibodies were purchased from Sigma-Aldrich (Gillingham, Dorset, United Kingdom).

### Cell Proliferation and Cytotoxicity Assays

AlamarBlue assay was used to assess cell proliferation ability. Briefly, 3 × 10^3^ cells (T47D) or 5 × 10^3^ cells (SK-BR-3) per well were seeded into a 96-well cell culture plate and incubated for 6 days. At the designated time points (Day 0, 2, 4, and 6), the culture medium was aspirated out and 100 μl of normal medium with 10 μl of the AlamarBlue reagent (Serotec Ltd., Oxford, United Kingdom) was added to each well. Cells were then incubated for 3 h at 37°C. The fluorescence signal was determined with a fluorescence plate reader (Promega, Southampton, United Kingdom) with excitation at 525 nm and emission at 590 nm. The percentage of proliferation during the incubation period was normalized with the fluorescence values at Day 0.

For the cytotoxicity assay, 1 × 10^4^ cells per well were loaded into a 96-well plate with a starving medium containing 1% FBS. After incubation overnight, the medium was changed with normal medium containing serial concentrations of paclitaxel (Sigma-Aldrich, United Kingdom) or cisplatin (Sigma-Aldrich, United Kingdom), respectively, and cultured for 48 h; cell cytotoxicity was then evaluated by the AlamarBlue assay described above.

### Scratch Wound Assay

Cells were loaded into a 24-well plate at a density of 2 × 10^5^ cells/well and cultured to reach confluence. The cell monolayer was then scratched with a 1 ml pipette tip to generate an artificial wound. After being washed twice with phosphate-buffered saline (PBS), 1 ml normal medium was added to each well. The migration of cells across the wound gap was monitored using an EVOS^®^ FL imaging system (Life Technologies, Carlsbad, CA, United States) with a 4× objective every 2 h for 48 h. The percentage of the gap closed area was measured and normalized by the data at hour 0 using the ImageJ software.

### Matrigel Invasion Assay

A transwell Matrigel assay was applied to evaluate the invasive ability of cells *in vitro*. Briefly, transwell inserts (8 μm pores) for a 24-well plate were pre-coated with 0.5 mg/ml Matrigel (BD Bioscience, Oxford, United Kingdom) for 1 h at 37°C. Subsequently, 2 × 10^5^ cells were loaded into the upper chamber in 150 μl of DMEM. The lower chamber was filled with 650 μl of normal medium. After incubation for 48 h, cells on the top side of the inserts were removed using a cotton swab. Chambers were fixed with 4% formalin for 30 min, and stained with 1% crystal violet for 30 min before rinsing with PBS. The number of invasive cells (underneath the inserts) was calculated by counting under a microscope (at least five counts per experimental setting).

### Cell-Matrix Adhesion Assay

A 96-well plate was pre-coated with Matrigel (10 μg/well) for 2 h at 37°C. 5 × 10^4^ cells were then added to each well and cultured for 4 h, followed by washing twice with PBS. Adhesive cells were fixed with 4% formalin and stained with 1% crystal violet. The number of adhesive cells was counted under a microscope (at least five counts per experimental setting).

### Patients and Specimens

The study protocol was approved by the Ethics Committee of Yantai Yuhuangding Hospital (Approval NO. 2018-109). Consecutive breast cancer patients (*n* = 22) who underwent neoadjuvant chemotherapy and surgery, from January 2017 to June 2017, were selected. All patients received ACx4 (Epirubicin and Cyclophosphamide) followed by Tx4 (Paclitaxel or Docetaxel) regimen for neoadjuvant chemotherapy. All patients recruited for this study were not pathological complete response after neoadjuvant chemotherapy in order to evaluate the differential expression level of S100P on tumor cells. During the same period, 10 cases of fibroadenoma were randomly selected as a control. Hormonal receptor status was determined by immunohistochemistry (IHC) described below. The HER2 status was examined following the American Society of Clinical Oncology guidelines. Based on the different combinations of ER, PR, HER2, and Ki67, patients were divided into four subgroups: luminal A, luminal B (luminal B HER2+ and luminal B HER2−), HER2+ and triple-negative.

### IHC

Formalin-fixed and paraffin-embedded tissue sections were freshly cut (4 μm) and mounted on silane-coated slides. After deparaffinization, the activity of endogenous peroxidase was blocked by exposure to 3% H_2_O_2_ for 5 min. All the sections were then boiled for 15 min at 250W in the Antigen Retrieval Solution (Dako Cytomation, Denmark). Non-specific binding was blocked with normal goat serum at room temperature for 20 min. Immunostaining was then carried out using the primary antibody S100P (1:100, Rabbit, #ab133554, Abcam) and incubated at 4°C overnight. After incubation for 25 min at room temperature using a biotinylated secondary antibody, slides were incubated with streptavidin–peroxidase complex (Avidin/Biotin Blocking Kit, #SP-2001, Vector Laboratories, Burlingame, CA, United States) for 25 min. Staining was visualized with 3,3’-Diaminobenzidine (DAB) and Mayer’s hematoxylin (1:10, Merck, Darmstadt, Germany) counterstain.

The intensity of S100P expression was evaluated by two qualified pathologists independently using a semi-quantitative scale of the Immuno Reactive Score (IRS). Briefly, the staining intensity was scored as 0 = none, 1 = weak, 2 = moderate, and 3 = high intensity. Additionally the percentage of positive stained cells was scored as 0 = no positive cells detectable, 1 = <10% of cells, 2 = 10–50% of cells, 3 = 51–80% of cells and 4 = >80% of cells. For the IRS, both scores were multiplied. The cases were divided into two groups showing no or weak staining (S100P: IRS < 4) and strong staining (S100P: IRS ≥ 4) (22).

### Statistical Analysis

Statistical analyses were performed using SPSS v25.0 (IBM Corporation, New York, NY, United States). Two-group comparisons were analyzed by a two-sided *t*-test when data were normally distributed, or Mann–Whitney U test when data were not normally distributed. Non-parametric test of two paired samples (Wilcoxon signed-rank test) or independent sample [Mann–Whitney or Kruskal–Wallis H(K)] was used to analyze the IHC results. Differences were defined as statistically significant when *p*-values were less than 0.05. *In vitro* experiments were repeated 2–4 times unless otherwise stated. The significance was shown in the figures as follows: ^∗^*p* < 0.05; ^∗∗^*p* < 0.01; ^∗∗∗^*p* < 0.001, and the *p* > 0.05 (no significance) was not shown.

## Results

### S100P Is Upregulated in Breast Cancer and Associated With Poor Prognosis

**FIGURE 1:**
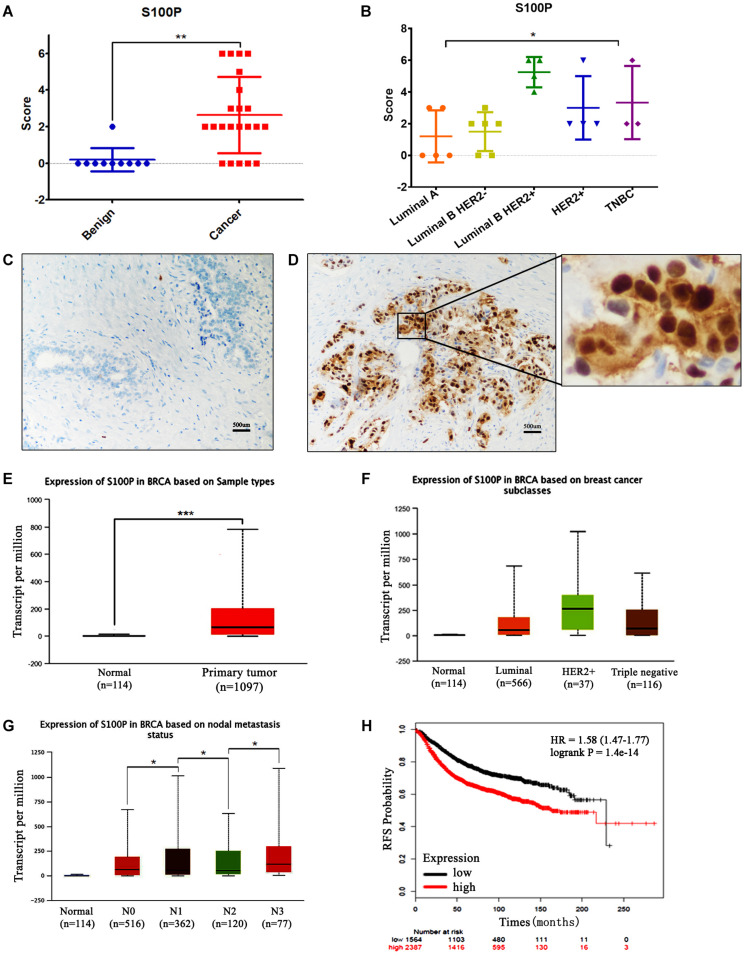
Expression levels of S100P in breast cancer and its association with survival. **(A)** IHC showed that expression levels of S100P in breast cancer were significantly higher than breast fibroadenoma (*p* < 0.001). **(B)** Expression levels of S100P in Luminal B HER2+ subtype breast cancer were higher than other subtypes (*p* = 0.041). **(C)** No S100P expression was observed in fibroadenoma tissue samples (IHCx200). **(D)** Strong S100P expression observed in breast cancer tissue samples (IHCx200). **(E)** S100P gene levels were significantly higher in breast cancer samples than in normal breast tissue, through the analysis of the TCGA breast cancer database (*p* < 0.001). **(F)** TCGA data showed that gene expression levels of S100P in HER2+ subtypes were higher than in other types. **(G)** TCGA data showed the gene expression levels of S100P in patients with lymph node metastasis (N3 > N2, N2 > N1, N1 > N0) (all *p* < 0.05). **(H)** KM-plotter database showed that the high expression level of S100P was associated with poor recurrence-free survival (RFS) in breast cancer patients (*p* < 0.001). *p < 0.05; **p < 0.01; ***p < 0.001.

The expression levels of S100P in 22 cases of breast cancer tissue samples before and after neoadjuvant chemotherapy, and 10 cases of breast fibroadenoma were detected by IHC. The results indicated that expression levels of S100P in breast cancer were higher than those in fibroadenoma (*p* < 0.001) (Figure 1A). Six out of twenty-two (27.27%) breast cancer patients showed strong staining of S100P, especially in Luminal B HER2+ subtype (4/4) (*p* = 0.041) (Figure 1B). In contrast, 9 out of 10 fibroadenoma patient samples did not show the expression of the S100P protein, with only one sample showing weak staining of S100P (Figure 1C). S100P protein was mainly located in the nucleus of the cells, with some cytoplasmic and cytomembrane staining (Figure 1D).

To verify our findings, we analyzed the TCGA breast cancer database and found that S100P gene expression levels in breast cancer tissue were higher than those in normal tissue (*p* < 0.001) (Figure 1E), which was consistent with our results. The expression levels of S100P in HER2+ breast cancer were higher than those in other subtypes (Figure 1F). S100P levels in patients with N3 lymph node metastasis was dramatically higher than that in N2 and normal tissues, and N2 > N1, N1 > N0 similarly (all *p* < 0.05, Figure 1G), which suggested that S100P may be involved in breast cancer metastasis.

We further used the KM plotter breast cancer database to explore the prognostic significance of S100P in breast cancer. As shown in Figure 1H, the high expression of S100P was closely associated with the poor recurrence-free survival (RFS) (*p* < 0.001) (Figure 1H) and overall survival (OS) (*p* < 0.001) (Supplementary Figure S1). For all different subtypes of breast cancer, high expression levels of S100P were all related to poor prognosis (data was not shown).

### S100P Increases Cell Proliferation and Adhesion *in vitro*

**FIGURE 2:**
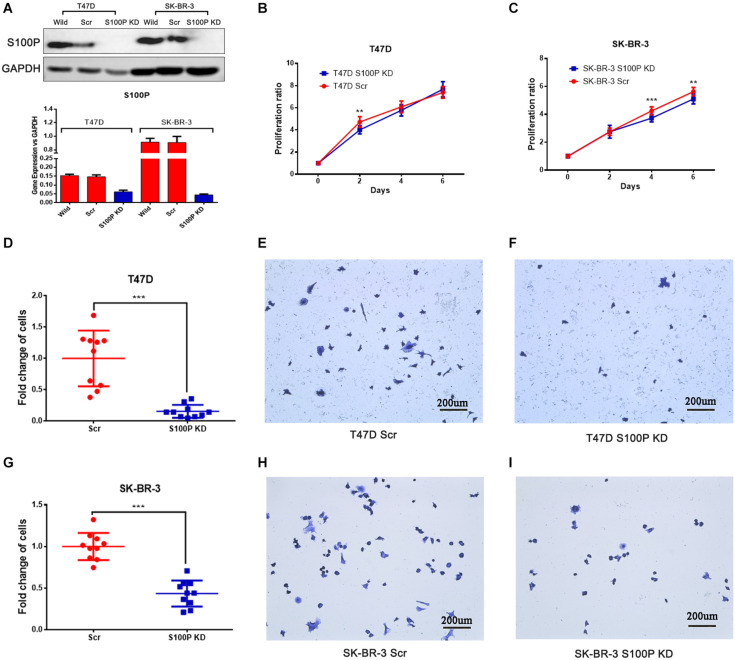
The effect of S100P on cell proliferation and adhesion in breast cancer. **(A)** S100P was knocked down in T47D and SK-BR-3 cells and validated by qRT-PCR and western blot. **(B)** S100P knocking down decreased cell proliferation in T47D cells (2 days, *p* < 0.01) (*n* = 10 per group). **(C)**. S100P knocking down decreased cell proliferation in SK-BR-3 cells (4 and 6 days, *p* < 0.01) (*n* = 10 per group). **(D–F)**. S100P knocking down reduced adhesive ability in T47D cells (*p* < 0.001). **(G–I)** S100P knocking down reduced adhesive ability in SK-BR-3 cells (*p* < 0.001). **p < 0.01; ***p < 0.001.

To investigate the function and mechanism of S100P in breast cancer, stable cell lines knocking down S100P by lentivirus siRNA transfection were established in T47D and SK-BR-3 cells. Assessed by qRT-PCR and western blot, levels of S100P were significantly decreased at both gene level (decreased at least 200%) and protein level (not detected) after S100P was knocked down (Figure 2A). This confirmed that stable cell lines with a low level of S100P were successfully constructed.

Cell proliferation assay was carried out *in vitro*, with results showing that the proliferative ability of T47D cells with a low level of S100P was significantly decreased by 15.04% (Day 2, *p* < 0.01, Figure 2B). Similarly, the proliferative ability of SK-BR-3 cells with low levels of S100P was decreased by 12.18% on Day 4 and 9.34% on Day 6 (*p* < 0.01, Figure 2C). These findings indicated that S100P promoted cell proliferation.

The effect of S100P on the adhesion of breast cancer cells was assessed by Matrigel adhesion assay *in vitro*. As shown in Figures 2D–I, the adhesive ability of breast cancer cells decreased in both T47D by 84.32% and SK-BR-3 cells by 56.51% after the knockdown of S100P (*p* < 0.001, respectively).

### S100P Increases Cell Invasion and Migration *in vitro*

**FIGURE 3:**
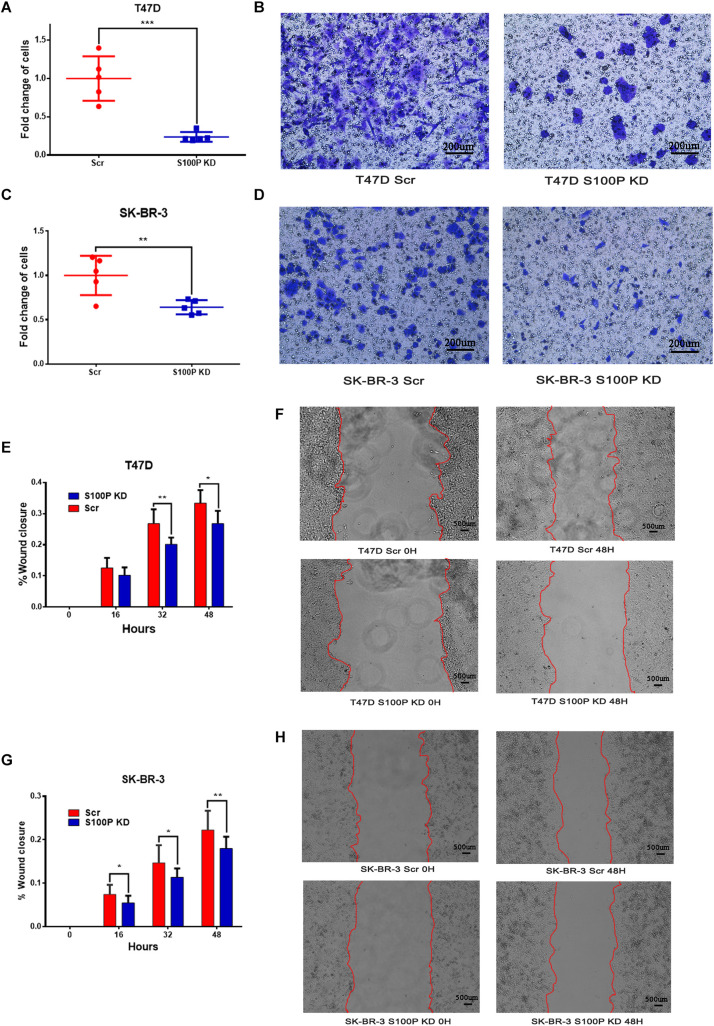
The effect of S100P on invasion and migration of breast cancer cells *in vitro*. **(A,B)** Invasive ability was significantly decreased in T47D cells after S100P was knocked down (*p* < 0.001). **(C,D)** Invasive ability was significantly decreased in SK-BR-3 cells after S100P was knocked down (*p* < 0.01). **(E,F)** Migrative ability was decreased in T47D cells after S100P was knocked down (48 h, *p* < 0.05) (*n* = 7 per group). **(G,H)** Migrative ability was decreased in SK-BR-3 cells after S100P was knocked down (48 h, *p* < 0.01) (*n* = 14 per group). *p < 0.05; **p < 0.01; ***p < 0.001.

To explore the influence of S100P on the invasion and migration of breast cancer cells, we conducted a transwell Matrigel assay and cell scratch experiment. As shown in Figures 3E–H, the invasive ability of breast cancer cells with low levels of S100P decreased significantly (T47D by 76.27%, *p* < 0.001; SK-BR-3 by 35.97%, *p* < 0.01) (Figures 3A–D). Similarly, the migration of breast cancer cells with low levels of S100P decreased significantly both in T47D and SK-BR-3 cells (T47D by 19.72%, 48 h, *p* < 0.05; SK-BR-3 by 19.16%, 48 h, *p* < 0.01).

### S100P Enhances the Chemosensitivity of Breast Cancer Cells

**FIGURE 4:**
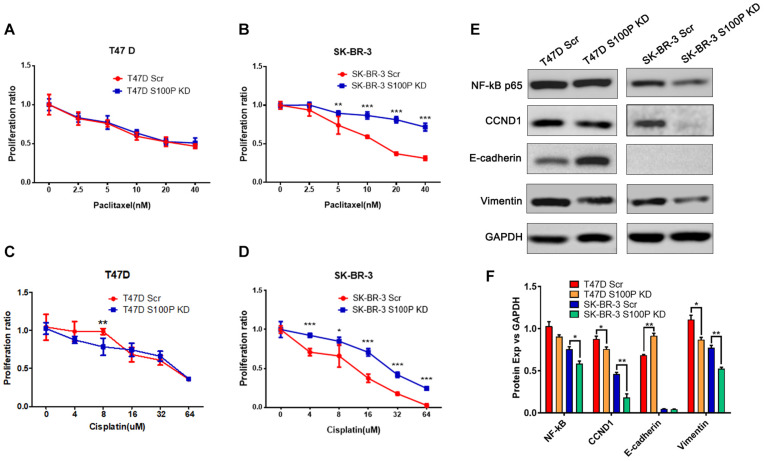
The effect of S100P on chemotherapeutic drug response in T47D and SK-BR-3 cells. **(A)**. S100P did not affect the paclitaxel sensitivity in T47D cells (*n* = 6 per group). **(B)**. SK-BR-3 S100P KD cells were significantly more resistant to paclitaxel (*n* = 6 per group). **(C)**. T47D S100P KD cells were more sensitive to low concentration cisplatin compared with the Scr control (8 μM, *p* < 0.01) (*n* = 6 per group). **(D)** SK-BR-3 S100P KD cells were more resistant to cisplatin compared with the Scr cells (*n* = 6 per group). **(E)** Protein levels of NF-κB, CCND1, E-cadherin and Vimentin assessed by western blot after S100P was knocked down in T47D and SK-BR-3 cells, respectively. **(F)** Quantitative densitometric analysis of western blots using ImageJ software. *p < 0.05; **p < 0.01; ***p < 0.001.

To explore the role of S100P in chemotherapeutic drug sensitivity in breast cancer, T47D (with Luminal HER2− subtype) and SK-BR-3 (with HER2+ subtype) cells were treated with different concentrations of paclitaxel and cisplatin, and cell viability was assessed by AlamarBlue assay. Results showed that expression levels of S100P did not affect paclitaxel sensitivity between T47D Scr and S100P KD cells (Figure 4A). However, at concentrations of 5 nM (*p* < 0.01), 10 nM (*p* < 0.001), 20 nM (*p* < 0.001), and 40 nM (*p* < 0.001), the SK-BR-3 S100P KD cells were significantly more resistant to the cytotoxic effects of paclitaxel than the Scr cells by 21.02, 46.62, 118.69, and 129.36%, respectively (Figure 4B). These results suggest that tumors with high expression levels of S100P are more sensitive to paclitaxel chemotherapy in HER2+ breast cancer cells (SK-BR-3).

Furthermore, we evaluated the effect of S100P on the chemosensitivity of breast cancer cells to cisplatin. As shown in Figure 4C, T47D S100P KD cells were more sensitive to low concentrations of cisplatin compared with T47D Scr cells by 18.09% (8 μm, *p* < 0.01), but there was no significant difference at high concentrations of cisplatin. For SK-BR-3 cells, the SK-BR-3 S100P KD cells were more resistant to cisplatin compared with Scr cells (4 μM by 30.51%, 16 μM by 89.67%, 32 μM by 139.49%, 64 μM by 898.97%, all *p* < 0.001; 8 μM by 28.77%, *p* < 0.05), indicating that the tumor with high S100P expression is more sensitive to cisplatin chemotherapy (Figure 4D).

### The Knockdown of S100P Alters the Expression of CCND1 and EMT-Associated Molecules

We conducted western blotting to unveil the mechanisms that S100P modulated the behaviors of breast cancer cells. As shown in Figures 4E,F, expression levels of the cyclin-D1 (CCND1) protein in T47D S100P KD cells and SK-BR-3 S100P KD cells were all decreased by 11.97 and 22.62% compared to their Scr controls. This suggests that S100P promoted cell proliferation by activating CCND1 protein in breast cancer.

We also detected the alteration of EMT related proteins after S100P was knocked down. Expression levels of E-cadherin in T47D S100P KD cells increased by 33.53% (Figures 4E,F), while expression levels of Vimentin decreased both in T47D S100P KD cells by 21.59% and SK-BR-3 S100P KD cells by 32.00%. These findings indicated that S100P increased cell migration and invasion by regulating E-cadherin and Vimentin.

### Levels of S100P Protein Is Reduced in Tumor Tissue After Chemotherapy

**FIGURE 5:**
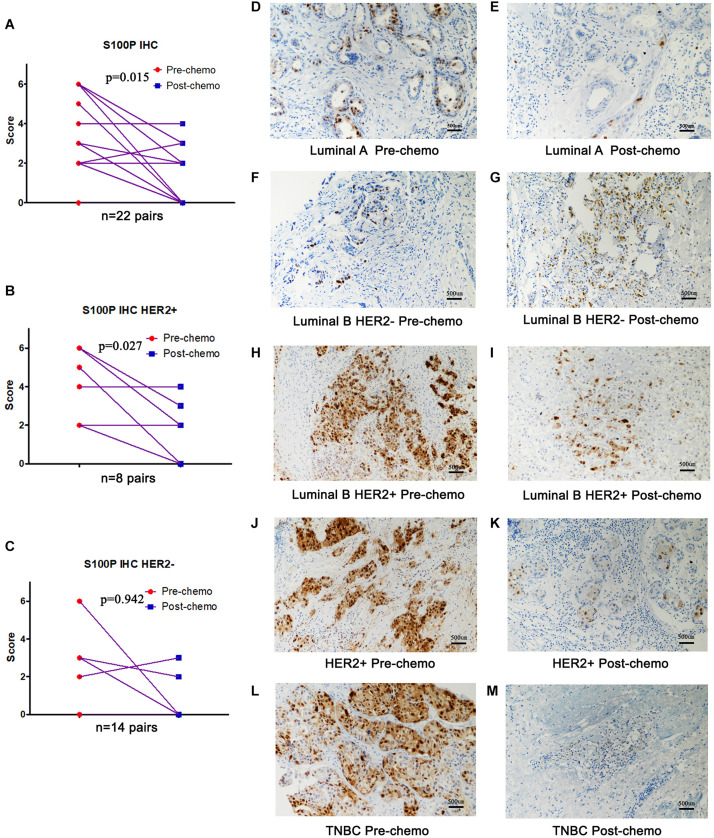
Expression levels of S100P in breast cancer tissue samples before and after neoadjuvant chemotherapy, detected by immunohistochemistry. **(A)** S100P levels in tumor tissue decreased significantly after chemotherapy (*p* = 0.015). **(B)** S100P levels in HER2+ tumor tissue decreased significantly after chemotherapy (*p* = 0.027). **(C)** The change of S100P levels in HER2 negative patients between pre-chemo and post-chemo was not significant (*p* = 0.942). **(D–M)** IHC of S100P expression pre-chemotherapy and post-chemotherapy in different subtypes of breast cancer patients (×200).

To verify the role of S100P in chemosensitivity of breast cancer cells, expression levels of S100P in 22 pairs of breast cancer tissue samples before and after neoadjuvant chemotherapy were examined by immunohistochemistry. This revealed that the expression levels of S100P in tumor tissue decreased by 46.55% after chemotherapy, compared with before chemotherapy (*p* = 0.015, Figure 5A). For subgroup analysis (Figures 5D–M), S100P in HER2+ tumor tissue decreased by 57.58% after chemotherapy (*p* = 0.027, Figure 5B), while there was no statistical difference in the change of S100P in the HER2− subgroup (*p* = 0.942, Figure 5C), which suggested that HER2+ breast cancer cells with higher levels of S100P were more sensitive to chemotherapy.

## Discussion

Metastasis and drug resistance are still tremendous challenges in breast cancer therapy leading to increased mortality. Therefore, identifying biomarkers of metastasis and elucidating mechanisms of drug resistance are of prime importance in breast cancer research.

Our results provide evidence that expression levels of S100P in breast cancer are significantly higher than those in fibroadenoma. The presence of S100P in the early stages of breast carcinogenesis suggests that S100P may serve as a biomarker to differentiate lesions at high risk of malignant evolution (19). Other studies have since reported that activation of HER2 increases the expression of S100P in breast cancer cells (16). The relation between S100P and HER2 overexpression is only significant for ER+ tumors (36), supporting that S100P may be associated with a hormone receptor-positive, HER2 enriched molecular subtype. In the present study, the expression levels of S100P is extremely high in Luminal B HER2+ breast cancer, which is supported by previous studies (36). This phenomenon may be more related to the “HER-enriched” breast cancer subtype (37, 38), suggesting that S100P may have a potential for the categorization of breast cancer and to be as a therapeutic target.

S100P is localized intracellularly in the nucleus, cytoplasm and cell membrane. C-terminally truncated form of S100P (t-S100P) is the major form of S100P and is exclusively located in the nucleus of breast cancer cells. High t-S100P is strongly prognostic for poor DFS, its efficacy confined to lymph node-positive tumors (39). Similar studies show patients with strong S100P nuclear expression have a significantly shorter OS and DFS (40, 41). S100P is prominent among genes upregulated in primary breast cancer cells with high-grade tumors (42). Likewise, its expression correlates to the level of the proliferative Ki-67 in primary breast cancer (17). The survival of breast cancer patients with S100P-positive cancers is significantly worse than those negative for S100P (18, 43). For TNBC patients, overexpression of S100P significantly correlates with more lymph node involvement, higher occurrence of metastasis and more recurrence events (22). Through analyzing the KM plotter breast cancer database, our data further confirms that high expression of S100P is closely associated with poor RFS and OS in breast cancer patients, which indicates that S100P exhibits a strong link with tumor progression and prognosis in breast cancer. On the other hand, the extracellular S100 proteins affect several intracellular signaling pathways through interacting with cytokines. The interaction of S100P and IFN-β exhibits suppressed cytotoxicity toward MCF-7 breast cancer cells, implying that the antitumor activity of IFN-β is suppressed by S100P. This mechanism could explain the S100P action as a factor which promotes tumor growth, angiogenesis, metastasis, drug resistance, and poor clinical outcome (44).

Previous studies indicate that S100P regulates some important proteins including CCND1 (45) and E-cadherin (46). Our study demonstrates that S100P enhances cell proliferation by activating CCND1, and promotes cell migration and invasion through downregulating E-cadherin and upregulating Vimentin in breast cancer *in vitro*. S100P is shown to promote cellular proliferation through binding with RAGE and activation of downstream molecules including ERKs, NF-κB, and β-catenin (47). S100P and Ezrin induce proliferation and migration in TNBC cells, and siRNA knockdown of Ezrin and S100P reduces the migration of these cells accompanied by an increased E-cadherin expression (23). Mechanistically, S100P induces EMT through binding to Integrin α7 and activation of the FAK (Focal Adhesion Kinase)/Src/Akt pathway, and upregulation of the expression of ZEB1 (46). S100P, Ezrin, and phospho-Ezrin^*Thr–*567^ are all involved in the transendothelial migration of TNBC cells and may act as potential targets in TNBC patients (23). Moreover, transfection of a vector expressing S100P into a benign, non-metastatic rat mammary cells induces a threefold increase in local muscle invasion and significant induction of metastasis in up to 75% of tumor-bearing animals, supporting the function of S100P as an inducer of breast cancer metastasis (43).

S100P not only promotes tumor progression and metastasis, but also plays a role in drug responses to chemotherapy, endocrine therapy, and targeted therapy. S100P levels may have a predictive value of response to chemotherapy, although findings are controversial between different cancer types. The implication of S100P in breast cancer chemotherapeutic drug response may be complicated. From our present study, the expression levels of S100P in tumor tissue decrease dramatically after chemotherapy compared with pre-chemotherapy, indicating S100P may have a chemo-sensitive role in breast cancer. *In vitro* experiments show the expression of S100P does not affect paclitaxel sensitivity in T47D cells (Luminal B HER2− subtype), while T47D S100P KD cells are more sensitive to low concentrations of cisplatin (e.g., 8 μM). In agreement with these results *in vitro*, there is no statistical difference in the change of S100P in HER2 negative breast cancer patients through analyzing the changes of S100P protein between pre-chemo and post-chemo breast tissues. This demonstrates that S100P does not play a significant role in chemotherapeutic drug response in HER2− breast cancer. However, in the HER2+ breast cancer cell line SK-BR-3, SK-BR-3 S100P KD cells are significantly resistant to paclitaxel and cisplatin compared with the vehicle control. This is confirmed in neoadjuvant chemotherapy patients that the expression level of S100P in HER2+ tumor tissues decreases significantly after chemotherapy compared with pre-chemotherapy. Therefore, our findings imply that S100P may have a role in predicting the drug sensitivity in HER2+ breast cancers. The different responses to chemotherapy might due to the different subtypes of breast cancer. However, the mechanism of this phenomenon remains unclear and requires further investigation to elucidate.

Resistance to hormone therapy is also a challenge for hormone receptor-positive breast cancers. ER+ breast cancer can escape antiestrogen treatment by up-regulating S100P (40). In a MCF-7 cell line with resistance to tamoxifen (TAM), the expression level of S100P is elevated. As the ER-regulated proliferation pathway is significantly suppressed after prolonged exposure to TAM, the S100P-RAGE signaling pathway via activation of ERK1/2 and NF-κB may be considered as a compensatory mechanism of cell proliferation and survival (40). Histone deacetylase 9 (HDAC9)-overexpressing cells are less sensitive to hydroxytamoxifen (OH-TAM) antiproliferative effects compared with parental MCF-7 cells through upregulating S100P (48). Therefore S100P may serve as a significant player in conferring acquired TAM resistance (40). Additionally, S100P is also involved in resistance to targeted therapies through activating the RAS/MEK/MAPK pathway to compensate for HER2 inhibition by trastuzumab, and inhibition of S100P leads to reversing the trastuzumab resistance (49). It remains unknown whether S100P is also involved in resistance to hormone therapies and targeted therapies in HR+ HER2+ breast cancers, which may be the direction of further investigation.

## Conclusion

In conclusion, S100P enhances the proliferation, adhesion, migration, and invasion of breast cancer cells through the regulation of NF-κB, CCND1, E-cadherin and Vimentin. S100P could be a promising therapeutic target in certain types of breast cancer. Additionally, S100P may be a biomarker to predict the therapeutic effects of chemotherapeutic agents in treating HER2+ breast cancer patients.

## Data Availability Statement

The original contributions presented in the study are included in the article/Supplementary Material, further inquiries can be directed to the corresponding author/s.

## Ethics Statement

The studies involving human participants were reviewed and approved by the Ethics Committee of Yantai Yuhuangding Hospital (Approval NO. 2018-109). The patients/participants provided their written informed consent to participate in this study.

## Author Contributions

YCo, GQ, and WJ: conceptualization. YCo and GQ: data curation. YCo and YCu: formal analysis, methodology, project administration, visualization, and writing – original draft. YCo: funding acquisition and software. YCo, YCu, SW, LJ, JC, SZ, and GQ: investigation. SZ, WJ, and YCu: resources. WJ and YCu: supervision. YCu: validation. EB, JL, FR, WJ, and YCu: writing – review, and editing. All authors provided critical feedback and helped to shape the research, analysis and manuscript.

## Conflict of Interest

The authors declare that the research was conducted in the absence of any commercial or financial relationships that could be construed as a potential conflict of interest.

## Supplementary Material

TABLE S1 The scores of IHC of S100P in breast cancer tissue samples before and after neoadjuvant chemotherapy.

FIGURE S1 Overall survival (OS) analysis of the S100P gene in breast cancer by KM plotting.

FIGURE S2 Representative images of the positive and blank controls of the IHC. Positive control and blank control of Immunohistochemistry. **(A)** positive control (human placenta tissue). **(B)** Blank control without S100P antibody (Human placenta tissue).
