# Supplementary material for: Calcium-Binding Protein S100P Promotes Tumor Progression but Enhances Chemosensitivity in Breast Cancer
Source: Front Oncol. 2020 Sep 15;10:566302. doi: 10.3389/fonc.2020.566302 (PMC7522638; doi:10.3389/fonc.2020.566302)
Supplement: TABLE S1 — The scores of IHC of S100P in breast cancer tissue samples before and after neoadjuvant chemotherapy. [file Table_1.DOCX]

Sup. Table 1.The S100P score of IHC

| **Case** | **Pre-chemo Score** | **Post-chemo Score** |
| --- | --- | --- |
| 1 | 0 | 0 |
| 2 | 0 | 0 |
| 3 | 0 | 0 |
| 4 | 3 | 0 |
| 5 | 6 | 3 |
| 6 | 5 | 0 |
| 7 | 2 | 0 |
| 8 | 2 | 0 |
| 9 | 2 | 3 |
| 10 | 6 | 0 |
| 11 | 2 | 3 |
| 12 | 0 | 0 |
| 13 | 2 | 2 |
| 14 | 2 | 3 |
| 15 | 0 | 0 |
| 16 | 6 | 3 |
| 17 | 2 | 3 |
| 18 | 6 | 2 |
| 19 | 3 | 0 |
| 20 | 3 | 2 |
| 21 | 2 | 3 |
| 22 | 4 | 4 |
