# Supplementary figures and images for: Calcium-Binding Protein S100P Promotes Tumor Progression but Enhances Chemosensitivity in Breast Cancer
Source: Front Oncol. 2020 Sep 15;10:566302. doi: 10.3389/fonc.2020.566302 (PMC7522638; doi:10.3389/fonc.2020.566302)

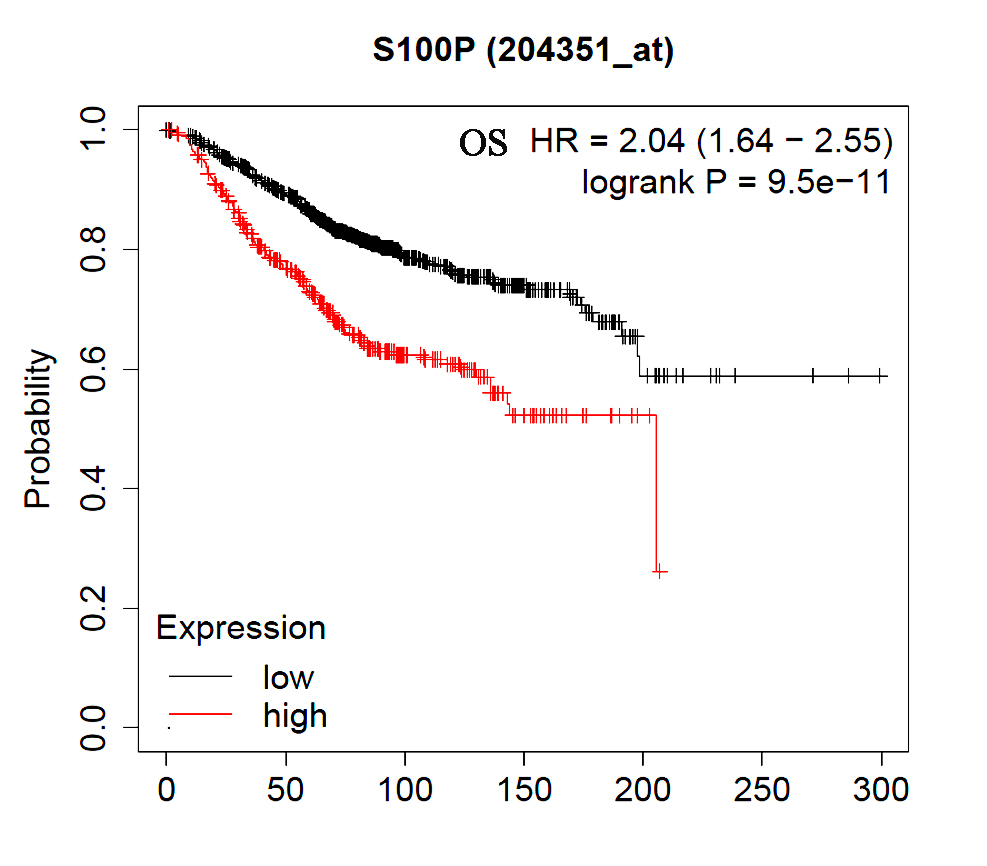

Supplement: FIGURE S1 — Overall survival (OS) analysis of the S100P gene in breast cancer by KM plotting. [file Image_1.JPEG]

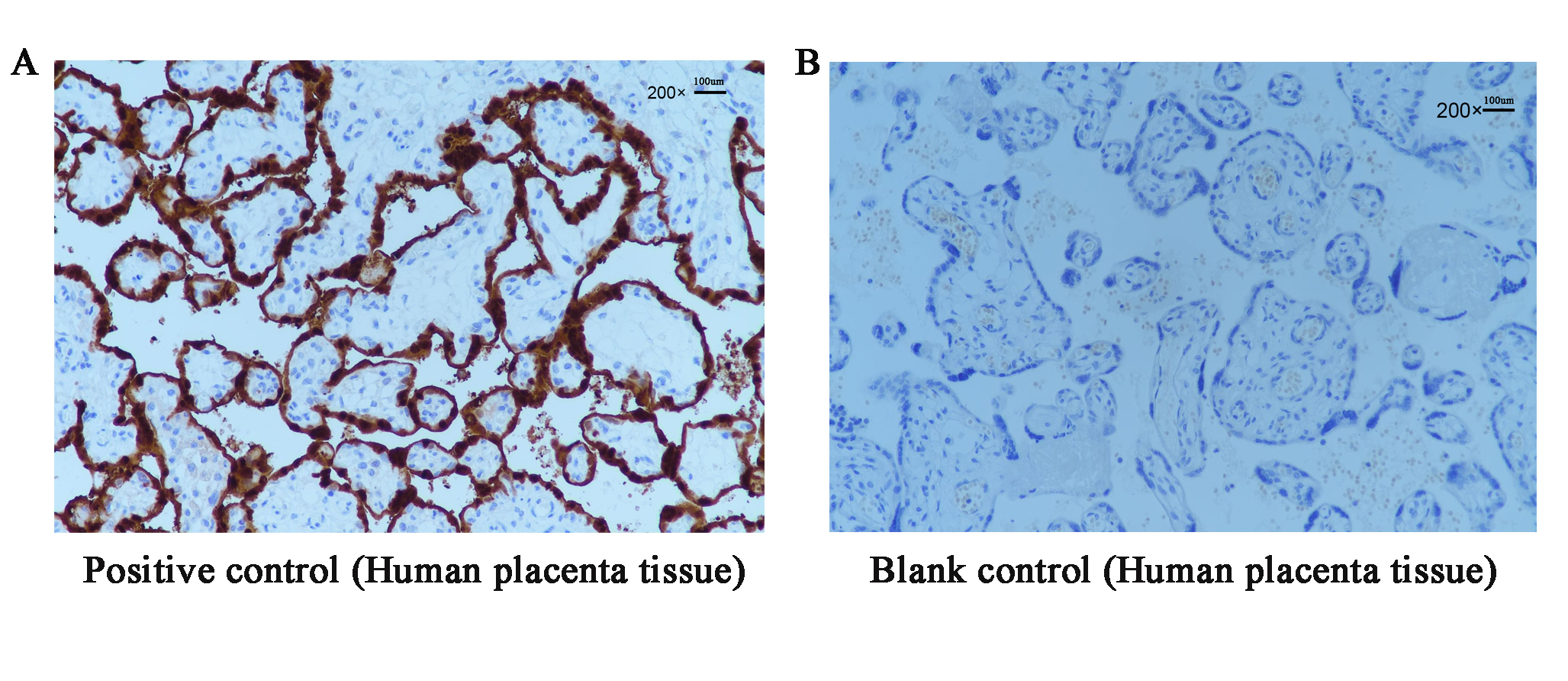

Supplement: FIGURE S2 — Representative images of the positive and blank controls of the IHC. Positive control and blank control of Immunohistochemistry. (A) positive control (human placenta tissue). (B) Blank control without S100P antibody (Human placenta tissue). [file Image_2.JPEG]
